# Silicon nanoparticles *vs* trace elements toxicity: *Modus operandi* and its omics bases

**DOI:** 10.3389/fpls.2024.1377964

**Published:** 2024-04-03

**Authors:** Mohammad Mukarram, Bilal Ahmad, Sadaf Choudhary, Alena Sliacka Konôpková, Daniel Kurjak, M. Masroor A. Khan, Alexander Lux

**Affiliations:** ^1^ Food and Plant Biology Group, Department of Plant Biology, School of Agriculture, Universidad de la Republica, Montevideo, Uruguay; ^2^ Department of Phytology, Faculty of Forestry, Technical University in Zvolen, Zvolen, Slovakia; ^3^ Plant Physiology Section, Department of Botany, Government Degree College for Women, Pulwama, Jammu and Kashmir, India; ^4^ Advance Plant Physiology Section, Department of Botany, Aligarh Muslim University, Aligarh, India; ^5^ Department of Integrated Forest and Landscape Protection, Faculty of Forestry, Technical University in Zvolen, Zvolen, Slovakia; ^6^ Institute of Forest Ecology, Slovak Academy of Sciences, Zvolen, Slovakia; ^7^ Department of Plant Physiology, Faculty of Natural Sciences, Comenius University in Bratislava, Bratislava, Slovakia; ^8^ Institute of Chemistry, Slovak Academy of Sciences, Bratislava, Slovakia

**Keywords:** silica, trace elements, metal stress, nanoparticles, heavy metal, oxidative stress, metalloid stress, sequestration

## Abstract

Phytotoxicity of trace elements (commonly misunderstood as ‘heavy metals’) includes impairment of functional groups of enzymes, photo-assembly, redox homeostasis, and nutrient status in higher plants. Silicon nanoparticles (SiNPs) can ameliorate trace element toxicity. We discuss SiNPs response against several essential (such as Cu, Ni, Mn, Mo, and Zn) and non-essential (including Cd, Pb, Hg, Al, Cr, Sb, Se, and As) trace elements. SiNPs hinder root uptake and transport of trace elements as the first line of defence. SiNPs charge plant antioxidant defence against trace elements-induced oxidative stress. The enrolment of SiNPs in gene expressions was also noticed on many occasions. These genes are associated with several anatomical and physiological phenomena, such as cell wall composition, photosynthesis, and metal uptake and transport. On this note, we dedicate the later sections of this review to support an enhanced understanding of SiNPs influence on the metabolomic, proteomic, and genomic profile of plants under trace elements toxicity.

## 1 Prologue: ‘heavy metals’ or ‘trace elements’: a terminological dilemma?

The term ‘heavy metal’ loosely signifies metals with a density higher than 7 g/cm^3^ (Bjerrum, 1936). The group supposedly enlist metals considered contaminants and can cause phytotoxicity or ecotoxicity *sensu lato*. However, there are several inconsistencies. Firstly, no authoritative list exists till now that notes all the heavy metals. Secondly, the ‘heaviness’ is somehow perceived as ‘toxicity’, which gave rise to anomalies such as including arsenic and antimony in this group even when they are not metals. Thirdly, density is neither a promising predictive factor when studying metal interaction with living organisms nor explains significant details about the element itself (Nieboer and Richardson, 1980). Thus, categorising them according to density is crude and non-scientific. Understandably, this classification has been refuted several times by plant scientists and others alike (Hodson, 2004; Appenroth, 2010). While Chapman (2007) amusingly suggests that the term would be better off with the ‘music’ industry rather than science, Duffus (2002) considers the word ‘meaningless’ and ‘misleading’. The IUPAC (International Union of Pure and Applied Chemistry) has neither recommended this term. It is unfortunate to witness the ever-increasing use of ‘heavy metals’ in the title and topic of refereed publications from several highly cited journals of plant and environmental science (see Pourret and Bollinger, 2017). It poses a moral dilemma for young researchers whether to use this term since the keyword ‘heavy metals’ still has massive indexing and visibility on scientific databases e.g., Web of Science and Scopus. Maybe it is what encourages the established research from the field to still use this misnomer (Cobbett and Goldsbrough, 2002; Rascio and Navari-Izzo, 2011; Ali et al., 2013; Pollard et al., 2014; Adrees et al., 2015).

Contrary to ‘heavy’ metals, other more appropriate and scientifically sound terms should be used to signify the characteristics and properties of the studied element. This could include ‘trace metals’, ‘toxic trace elements’, or ‘potentially toxic trace elements’ in perspective research. ‘Trace elements’ are those elements ‘found in low concentration, in mass fractions of ppm or less, in some specified source, e.g., soil, plant, tissue, groundwater, etc.’ (Duffus, 2002). However, referring to these elements or metals as toxic is imprecise again or redundant at best. Paracelsus (1493-1541) laid the fundamental rule of toxicology: all elements and their derivatives are toxic in high enough doses (see Duffus, 2002). Therefore, we recommend the usage of ‘trace elements’ in the title and as a topic for future studies related to toxic trace elements. We also urge the responsible authorities, particularly editorial board members, to discourage the usage of the ‘heavy metals’ keyword in future submissions.

## 2 Introduction

A plant’s health chiefly depends on soil composition. Soils have frequently been exposed to excessive amounts of essential and non-essential nutrients through industrial wastes, municipal composts, agricultural effluents, sewage sludge and surface mining wastes, and their toxic levels damage plant species differently (DalCorso, 2012). Trace elements (TEs) are a group of elements present in low concentration (mass fraction of ppm or less) in the specified medium (soil, plant, etc.) and includes Cd, Pb, Mn, As, Fe, Cr, Cu, Ni, Co, Ag, Zn, Sb, Ti, and Hg. TEs contamination has become a severe environmental threat worldwide. Besides naturally deriving from parent rocks, most of the TEs in the soils result from anthropogenic activities such as mining and processing of metal ores, energy and fuel production, intensive agriculture, and sewage processing, including several other industrial processes (Tóth et al., 2000; Khan et al., 2008; Brunetti et al., 2009; Yadav et al., 2019). TEs are not bio- or thermo-degradable so that they may persist in the soil for thousands of years, given their relative non-mobility and their technically and financially demanding remediation from the soil (Rahimi et al., 2017; Masindi and Muedi, 2018). Natural soils are the primary source of TEs in plants. Despite the selective membrane of root cells, much of the elements present in the soil translocate into plant tissues. In contrast, their availability depends mainly on the solubility in the soil solution or the root exudates (Blaylock and Huang, 2000). Therefore, the plants may efficiently uptake hazardous TE levels, affecting their functioning and animal and human health through the food chain (Adriano, 2001).

Some trace elements such as Zn, Cu, Ni, Fe, Mo, and Mn are essential for plant metabolism. Zn has been shown to play a crucial role in enzyme systems involved in carbohydrate and protein metabolism, auxin formation, and stabilises cell membrane integrity (Hafeez, 2013). There is also evidence that Zn may contribute to the plant defence system by regulating stress protein expression and stimulating the antioxidant enzymes (Cabot et al., 2019; Hassan et al., 2020). Ni has been reported as an integral component of various enzymes essential for ureolysis, nitrogen fixation, hydrogen metabolism, and antioxidant system (Fabiano et al., 2015; Lavres et al., 2016; Siqueira Freitas et al., 2018). Similarly, Fe forms cofactors of many vital enzymes and is a central component of the electron transport chain and a crucial element for chlorophyll biosynthesis (Schmidt et al., 2020). Cu plays a pivotal role in regulating the photosynthetic and respiratory electron transport chain, besides affecting cell wall formation, antioxidant activities, and hormone perception (Yamasaki et al., 2008; Printz et al., 2016). Furthermore, Mn is crucial for photosynthetic machinery as the primary cofactor for the oxygen-evolving complex in photosystem II (PSII) and may participate in plant antioxidative system (Millaleo et al., 2010; Alejandro et al., 2020). Besides the earlier mentioned TEs, several studies proved the beneficial role of Co and Cr for plant growth and yield, although they are not classified as essential nutrients (Samantaray et al., 1998; Gad, 2012; Akeel and Jahan, 2020). On the contrary, TEs such as Pb, Cd, Hg, and As have no documented beneficial role in the metabolism of higher plants. They are considered the “main threats” even in trace amounts (Chibuike and Obiora, 2014). The effect of TEs toxicity depends, of course, on a particular element involved in the process and its concentration in the soil. However, it may vary significantly among plant species and varieties. Such variations result from the different (i) pathways and mechanics through which TEs are absorbed by roots (Williams et al., 2000), (ii) mechanisms of their releasing and redistribution into the shoot, and (iii) abilities to exclude, chelate or accumulate TEs in particular structures, which plants have adopted (Salt et al., 1998). These mechanisms are involved in the maintenance of essential TEs homeostasis. Furthermore, the plant species can be divided into (hyper)accumulating and non-accumulating plants, whereas most of the plant kingdom is considered non-accumulators (Viehweger, 2014). However, in general, TEs toxicity leads to the blocking of functional groups of many enzymes (Tang et al., 2020), malfunctions in photosynthetic machinery (Giannakoula et al., 2021), production of reactive oxygen species (ROS) and associated oxidative damage (Ma et al., 2022; Sardar et al., 2022), and impairment of plant mineral nutrition through the replacement of essential nutrients at cation exchange sites of plants (Arif et al., 2016). Such alterations in overall biochemistry and physiology affect plant development and growth and may lead to plant death in severe cases (Chibuike and Obiora, 2014).

To date, only a handful of published articles target the interaction of TEs with silicon nanoparticles (SiNPs). The existing reviews on this nexus deal mostly with heavy metals, the term which is in itself confusing (*vide supra* section Prologue), and therefore, several toxic elements were purposefully left behind. Also, the existing literature reviews often need to restrict their significant discussion to SiNPs over bulk silicon or address the omics aspect sufficiently. In our earlier review article, we demonstrated SiNPs potential in mitigating the abiotic stress in general, where heavy metal stress was also discussed (Mukarram et al., 2022). Nonetheless, one of this article’s limitations was the absence of an elaborated mechanism on SiNPs dialogue with TEs toxicity.

To overcome these concerns, we included a wide range of toxic elements in the present review that were studied with SiNPs, irrespective of their ‘heavy metals’ stigma. We also addressed how SiNPs could interact with plant metabolomics, proteomics, and genomics during TEs toxicity. So, the novelty of this review article over the existing ones lies in its understanding of the SiNPs-TEs interaction and its omics perspective. Through this article, we hope to instigate a discussion among the silicon community regarding its active correspondence with plant physiology, especially when there are still several ambiguities around this nexus.

## 3 Trace elements phytotoxicity

**Figure 1:**
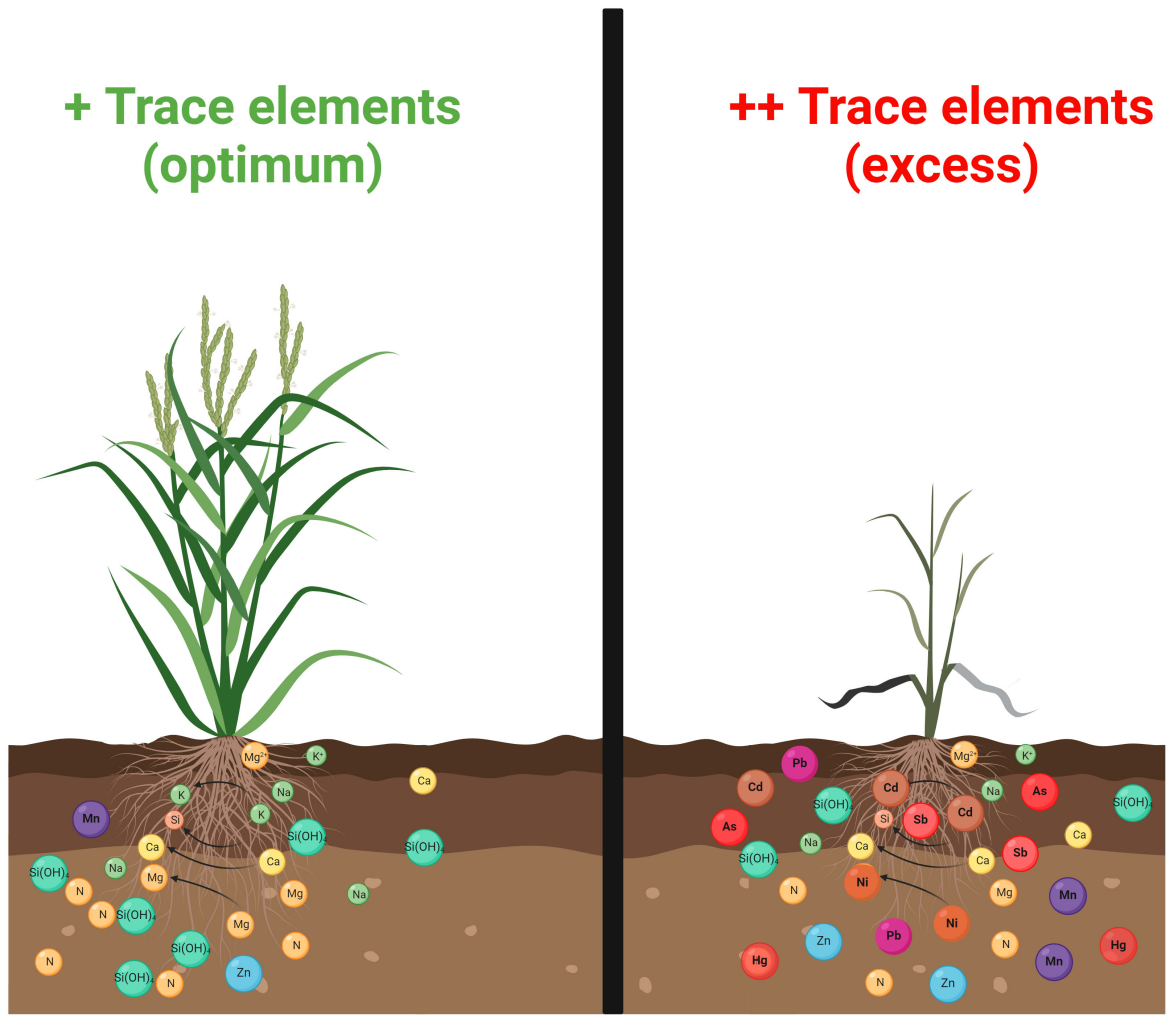
Trace elements (TEs) phytotoxicity in higher plants. The optimum concentration of several TEs promotes plant growth and development (such as Zn, Cu, Ni, Fe, Mo, Mn, Cu, and Cr) (panel on the left). Nonetheless, their overaccumulation, in addition to other toxic elements (such as Hg, As, Cd, and Pb), jeopardises cellular homeostasis and retard plant physiology and productivity (panel on the right).

Although several TEs are essential to plants, their overaccumulation in agricultural soils endangers plant growth and development while compromising crop marketability and global food security (Asati et al., 2016; **Figure 1**). Variations in responses of different plant species to TEs toxicity have been observed. Plant behaviour can change with soil pH and composition and specific TEs. TEs toxicity potentially alters root and shoot morphology and anatomy (Martinka et al., 2014). It adversely affects photosynthesis and respiration by changing the leaf’s structural integrity and physiology, damaging energy (photon) allocation, and regulating critical metabolic processes (Küpper et al., 2002; Ying et al., 2010; Chandra and Kang, 2016). Hampered growth, chlorosis, necrosis, changes in stomatal functions, leaf rolling, lowered water potential, altered membrane function, efflux of cations, and changes in the activities of critical metabolic enzymes are the widely reported symptoms of TEs toxicity in plants (Van Assche and Clijsters, 1990; Marschner, 2012; Hasan et al., 2017; Aponte et al., 2020). TEs toxicity severely affects PSI and PSII, restricting photosynthetic output. TEs accumulation targets two crucial photosynthetic enzymes, i.e., ribulose 1,5-bisphosphate carboxylase (RuBisCO) and phosphoenol pyruvate carboxylase. Cd has been reported to alter the structure and activity of RuBisCO by substituting Mg^++^ ions, which are needed as a cofactor of carboxylation reactions (Ashfaque et al., 2016). At the cellular level, these TEs cause configurational changes in the endoplasmic reticulum, Golgi apparatus, chloroplast, and mitochondria and increase nucleus size and cellular vacuolisation (Małkowski et al., 2019; Sperdouli et al., 2022; Liu et al., 2023). A rise in oxidative stress linked with excessive accumulation of TE ions is strongly considered the first symptom of TE-induced toxicity (Rodríguez-Serrano et al., 2009; Sharma and Dietz, 2009; Ghori et al., 2019; Gupta et al., 2019; Ma et al., 2022). To cope with the oxidative stress caused by TEs toxicity and protect cellular and subcellular compartments, plants have developed several mechanisms to sustain the essential TEs ion concentrations and lessen exposure to non-essential TEs. Among these tolerance mechanisms, some are required for metal homeostasis that lowers the damage via exclusion, detoxification, the restriction of metal ions into the apoplast, and the extracellular chelation of metal ions (Ovečka and Takáč, 2014). However, other mechanisms involve extruding individual TE ions from the intracellular environment or their sequestration into the compartments to separate them from other important cellular components (Manara, 2012). Hyper-tolerance and the hyperaccumulation of TEs in the plant body without having any harmful effect on viability are the best-known strategies employed by plants under a TEs-induced toxic environment (Baker and Brooks, 1989; Assunção et al., 2001, Assunção et al., 2003; Vaculík et al., 2009, Vaculík et al., 2012; Van der Ent et al., 2013; Baker et al., 2020; Pinto Irish et al., 2023).

## 4 SiNPs and plants: uptake and action during the optimal environment

Although biogenic silicon (bulk-Si) is classified as a non-essential element for plant growth and development, its stress-mitigating potential has been widely reported (Korndörfer and Lepsch, 2001; Ma, 2004; Liang et al., 2015; Luyckx et al., 2017; Vaculík et al., 2020). Silicon is the most abundant metalloid on the earth’s surface. However, most Si is present in the soil as insoluble oxides or silicates, which is unavailable for plant uptake. The chemical weathering of silicate minerals liberates dissolved Si as plant-available monosilicic acid, whereas its concentration in soil solution commonly varies between 0.1 and 0.6 mM (Epstein, 1994; Hodson et al., 2005). In this context, recent advances in nanotechnology could alleviate the scarfed amount of monosilicic acid in most cultivated soil and the limits of silicate fertilisation via the connection of Si-derived benefits with benefits associated with the properties of nanoparticles (Bhat et al., 2021). The smaller size and broader absorption surface area of SiNPs over bulk-Si should enable their easier absorption, distribution, and accumulation in plants (Galbraith, 2007). However, Etxeberria et al. (2009) consider nanoparticle uptake an active transport requiring various other cellular mechanisms such as recycling, signalling, and regulating the plasma membrane.

**Figure 2:**
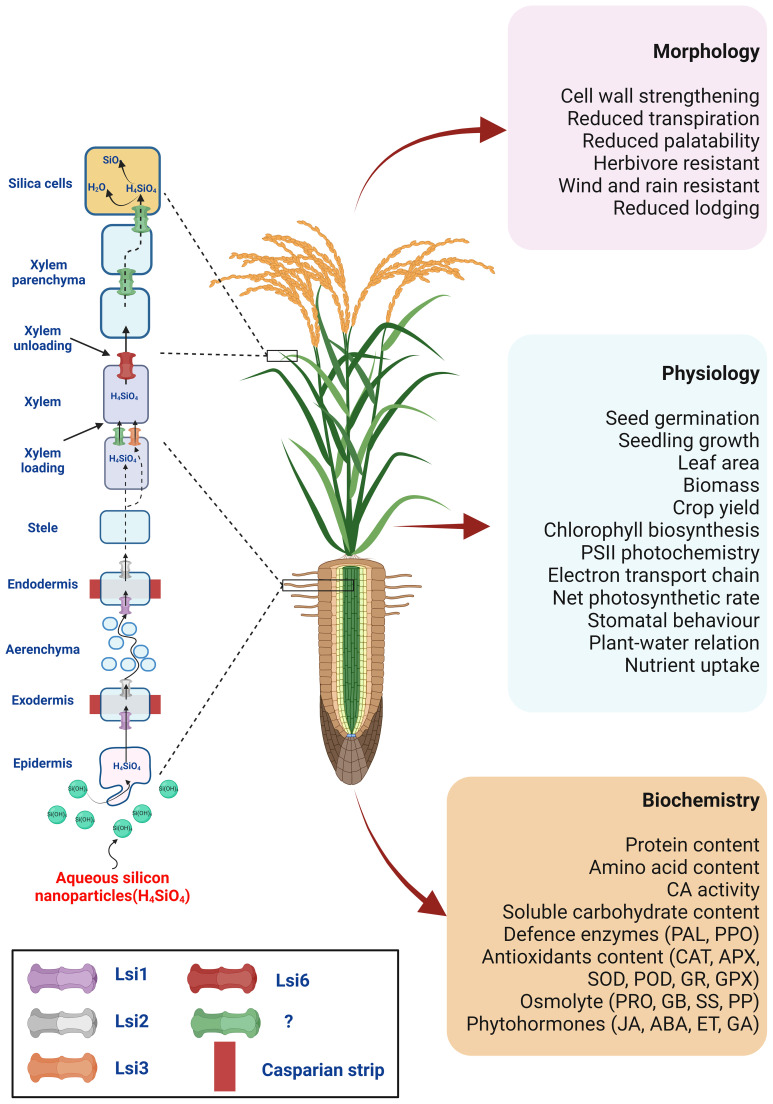
The hypothetical model for SiNPs uptake, transport, and action in higher plants under physiological settings. It is possible that SiNPs, like bulk silicates, could be absorbed by plant roots in the form of silicic acid (Si(OH)_4_) and transported to endodermis by aquaporin channel Lsi1. Lsi2 might facilitate xylem loading at the endodermis-stellar apoplast junction. From there, it could join the transpiration stream to move to aerial parts. Lsi6 could assist xylem unloading at the shoot for distribution to shoot tissues or deposition in the cell walls or as specified silica cells (phytoliths). Silica deposition at the cell wall, silica cells or phytoliths are crucial to cell wall strengthening, reduced palatability for herbivores, and resisting wind, rain, and lodging. This action can be understood as the ‘direct’ effects of SiNPs. Additionally, SiNPs supplementation shows a strong correlation with superior plant physiology. This includes improved seed germination and seedling development, photosynthesis, gas-exchange, plant-water relation, nutrient uptake, and redox homeostasis. The direct involvement of SiNPs in these upgrades still lacks unequivocal proof. Nonetheless, several research findings support the possible ‘indirect’ interaction of SiNPs with plant biochemistry and physiology. These mechanical and physiological enhancements mediate SiNPs-induced growth and productivity in higher plants.

Despite a scarcity of available reports, Mukarram et al. (2022) discussed the SiNPs could follow a similar transport route as their bulk counterpart – the plant root absorbs Si from the soil solution in the form of monosilicic acid (Si(OH)_4_) (Mitani-Ueno and Ma, 2021). The absorption and distribution of Si in the plant are ensured by two different types of Si transporters: channel-type transporters (referred to as Low Silicon 1, Lsi1) and efflux transporters (referred to as Low Silicon 2, Lsi2), which were first described in rice (Ma et al., 2006, Ma et al., 2007). The Lsi1 from the nodulin-26 major intrinsic protein (NIP) III subgroup of aquaporins drives the passive influx of Si from the apoplast into the root cells. At the same time, Lsi2, belonging to an uncharacterised anion transporter family, is responsible for the active efflux of Si from the root cells towards the xylem, i.e., xylem loading (Ma and Yamaji, 2015). Following monosilicic acid absorption in the root stele, Si is transported to the shoot through the xylem by transpirational flow, with subsequent Si unloading to the leaf epidermal cells. As the content of monosilicic acid in the cells increases, monosilicic acid becomes highly polymerised and changes to form an amorphous silica gel (SiO_2_·nH_2_O) (Mitani et al., 2005; Schaller et al., 2021; de Tombeur et al., 2022). The silica can accumulate under the epidermal cell wall, forming cuticle-silica double layers which provide additional protection against mechanical injury and fungal, bacterial, nematode, and insect attacks (Debona et al., 2017; Rastogi et al., 2019; Zellner et al., 2021). The transporter ensuring the xylem loading is not yet fully known undoubtedly. However, Yamaji et al. (2008) described the Lsi6 transporter in rice, which is responsible for the Si unloading from the xylem and subsequently regulating its deposition in the shoots (**Figure 2**).

All abovementioned transporters are localised in the plasma membrane; however, they show different tissue and/or cellular specificity of their localisation, indicating that they are involved in different steps of absorption, xylem loading, and distribution of Si (Ma et al., 2011; Ma and Yamaji, 2015). Besides, interspecific differences in the presence, tissue, cellular localisation, and polarity of transporters, as well as their expression patterns, exist, determining the different abilities to accumulate Si in various plant species (Ma et al., 2011; Ma and Yamaji, 2015; Mitani-Ueno and Ma, 2021). Accordingly, the plant species are divided into accumulators, intermediate accumulators, and non-accumulators (Takahashi et al., 1990). The Poaceae, Equisetaceae and Cyperaceae families are known accumulators (>4% Si), the Cucurbitales, Urticales and Commelinaceae intermediate Si accumulators (2–4% Si), while most of the other species have little or no ability to accumulate Si (Hodson et al., 2005; Currie and Perry, 2007).

SiNPs involvement with several metabolic and physiological activities has been described under optimal and stress conditions (El-Shetehy et al., 2021; Fan et al., 2022; Naaz et al., 2023). SiNPs can improve photosynthesis by PSII reaction centres opening and promoting the absorption, transmission, and transformation of light energy, the electron transport rate of PSII, chlorophyll and carotenoid biosynthesis, and other related enzymes (Sharifi-Rad et al., 2016; Fatemi et al., 2020; Mukarram et al., 2021). Moreover, SiNPs can upregulate the expression of many genes encoding proteins directly involved in photosynthetic machinery (Song et al., 2014; Hassan et al., 2021). The smaller sized-SiNPs can penetrate seed coat promptly and improve seed germination and growth and later overall growth, development, and crop yield (Epstein, 1994; Haghighi et al., 2012; Azimi et al., 2014; Janmohammadi et al., 2016; Karunakaran et al., 2016; Sun et al., 2016; Kheyri et al., 2019). In addition, SiNPs can trigger the multiplication of growth-promoting rhizobacteria responsible for nutrient recycling and soil health maintenance, promoting plant maturation (Karunakaran et al., 2013). All these functions contribute to plant resistance against various physical, chemical, and biological stressors (**Figure 2**).

## 5 SiNPs-mediated TEs sequestration

The TEs sequestration from plant environs, such as soil, air, and water, is a constant challenge. Several experiments have recently targeted sustainable approaches to remedy TEs excess. Among various methods, the application of SiNPs in the form of a foliar spray, seed priming, and soil incorporation has emerged as a novel and eco-friendly approach to combat TEs stress (Asgari et al., 2018; Hussain et al., 2019; Rizwan et al., 2019). SiNPs treatment effectively enhanced the photosynthesis and growth in plants exposed to TEs-stressed conditions (Cui et al., 2017; Khan et al., 2020a). At the latest, the magnetic properties of SiNPs are curative towards contaminated water (Mahboub et al., 2022). SiNPs might operate in several ways to sequestrate different TEs, such as forming complexes with toxic TE ions, arresting their uptake, TEs compartmentalisation within plants, stimulating the antioxidant defence system, and other omics aspects (Kopittke et al., 2012; Lukačová et al., 2013; Tripathi et al., 2016; Zhou et al., 2021). We will explore these possibilities in the later sections.

### 5.1 Cadmium

Being a non-essential trace element, the accumulation of Cd in agricultural soils is an onerous threat to plants (Haider et al., 2021). Thus, eliminating Cd from the soil is crucial to sustaining food security and environmental safety. SiNPs-mediated amelioration of Cd has been reported in several plant species, including *Phyllostachys edulis* (Emamverdian et al., 2021), *Satureja hortensis* (Memari-Tabrizi et al., 2021), *Oryza sativa* (Cui et al., 2017; Hussain et al., 2020), and *Triticum aestivum* (Hussain et al., 2019). Applying SiNPs against Cd stress has been considered more efficient than regular fertilisers (Chen et al., 2018). The experiment on *Phyllostachys edulis* suggested that SiNPs make a complex with Cd ions via adsorption and reduce the accumulation of Cd in roots and leaves (Emamverdian et al., 2021). It subsequently enhances the germination and growth parameters. Soil-applied SiNPs can alleviate Cd stress in *Triticum aestivum* plants with improved growth and chlorophyll content (Ali et al., 2019; Khan et al., 2020a). Further, SiNPs minimise Cd accumulation and oxidative stress while improving nutrient uptake and antioxidant defence system in *Triticum aestivum* (Thind et al., 2021). Roots treated with SiNPs have increased the xylem cell wall lignification in *Trigonella foenum-graceum* (Nazaralian et al., 2017). The increased cell wall lignification was coupled with an improved xylem cell wall thickness. Such cell wall adjustments improve nutrient transport and silicon for faster growth (Asgari et al., 2018). SiNPs engage the Cd ions on the root surface to terminate their translocation in the aerial parts or immobilise them in the soil (Silva et al., 2017). Cui et al. (2017) also reported the downregulation of *OsLCT1* and *OsNramp5*, genes with SiNPs application involved in Cd uptake and transport, respectively, in *Oryza sativa*. At the same time, SiNPs upregulated genes involved in Cd transport into the vacuole (*OsHMA3*) and Si uptake (*OsLsi1*). Higher silicon uptake can further restrict Cd uptake and transport and, thus, Cd toxicity.

### 5.2 Lead

Pb is a non-essential element and a detrimental contaminant for agricultural soils. It hampers plant metabolism, cell adhesion, and signalling by accumulating ROS in the cell wall (Küpper, 2017; Aslam et al., 2021). Foliar application of SiNPs boosted photosynthetic machinery and antioxidant enzymes in *Coriandrum sativum* and restricted Pb toxicity (Fatemi et al., 2020). A tissue culture experiment on Pb stress mitigation via silicon dioxide nanoparticles in *Pleioblastus pygmaeus* showed a reduction in the soluble protein content assimilated in the cell membrane while maintaining the cell membrane vitality (Emamverdian et al., 2019).

### 5.3 Arsenic

Arsenic contaminates groundwater globally, and irrigation with As-rich water amplifies its bioaccumulation and toxicity in several crops (Finnegan and Chen, 2012; Farooq et al., 2016; Vaculík and Vaculíková, 2017; Abbas et al., 2018; Abedi and Mojiri, 2020). Nonetheless, SiNPs application arrests As uptake and translocation to aerial parts of tissues and alleviates phytotoxicity in *Solanum lycopersicum* (González-Moscoso et al., 2019, González-Moscoso et al., 2022). SiNPs were reported to lower oxidative stress via improving antioxidative defence (SOD, APX, GR, and DHAR) in *Zea mays* seedlings (Tripathi et al., 2016). Further, SiNPs increase the mechanical strength of the cell wall in rice suspension cells under As toxicity via increasing pectin content, cation exchange capacity, and pectin methyl-esterase activity, reducing pectin methyl-esterification. SiNPs also blocked the uptake of As by inhibiting the expression of genes encoding As uptake (*OsLSi* 1, low silicon 1; *OsLSi* 2, low silicon 2) (Cui et al., 2020) since As and Si share a common transport system. Thus, adding SiNPs into the As medium causes a direct competition for the transport proteins (Cui et al., 2020). Moreover, SiNPs treatment enhances the expression levels of plasma-membrane localised NIP aquaporin family proteins, *OsNIP1;1* and *OsNIP3;3*, which are permeable to arsenite (Mitani-Ueno et al., 2011; Sun et al., 2018). The overexpressed *OsNIP1;1* and *OsNIP3;3* are reported to reduce the As accumulation in *Oryza sativa* plants (Sun et al., 2018).

### 5.4 Mercury

Mercury (Hg) is a highly toxic pollutant that contaminates cropland to different extents worldwide (Liu et al., 2020a). Li et al. (2020) demonstrated that exogenously applied SiNPs ameliorate the adverse effects of Hg in *Glycine max* seedlings. SiNPs significantly reduced Hg uptake, accumulation, and translocation in these seedlings. Further analysis with synchrotron radiation X-ray fluorescence showed lower Hg accumulation in the epidermis and pericycle of roots and stems of *Glycine max* plants treated with SiNPs.

### 5.5 Chromium

SiNPs-mediated alleviation of Cr toxicity has been reported in *Pisum sativum* via reduced Cr uptake and accumulation in plant tissues (Tripathi et al., 2015). It was proposed that SiNPs facilitated mineral nutrient uptake and downplayed ROS synthesis by triggering antioxidant enzymes. SiNPs protected leaf ultrastructure under Cr toxicity in *Triticum aestivum* (Manzoor et al., 2022). While Cr deteriorated cellular organelles, SiNPs protected the cell walls, cell membranes, mitochondria, granal lamellae, thylakoids, nucleoli, and nuclear membrane. In a hydroponic study with *Brassica napus*, Huang et al., 2024 witnessed 100 um SiNPs (20 nm) boosted Si content in leaves increased by 169%, mostly restricted to intercellular spaces, chloroplasts, guard cells, and stomata. This can upgrade PSII biochemistry (NPQ, ETR, and quantum yield of PSII) and photosynthetic productivity. In the same study, SiNPs hampered the expression of Cr (and related TEs) transporter genes such as *ST1*, *ST8*, *ABCG37*, *HMA*, and *MT*, resulting in decreased Cr uptake (by 92% in roots and 76% in leaves). In *Oryza sativa* seedlings, SiNPs reversed the Cr-induced cell cycle arrest at the G2/M phase along with IAA application (Sharma et al., 2022). Similarly, endogenous NO levels in root tips were improved which could assist in ROS scavenging and upregulated antioxidant activity as was reported in the study.

### 5.6 Copper

Emamverdian et al. (2020) demonstrated the mitigative effect of SiNPs on three different TEs stresses: Mn, Cu, and Cd on *Arudinaria pygmaea*. Enhanced localisation of Cu and Mn by SiNPs was observed in the root surface, which could minimise TEs accumulation in the stem and leaves. Also, SiNPs treatment enhanced the photosynthetic capacity, biomass, and overall growth, which authors correlated with the reduced TEs uptake and accumulation in the plant shoot. Similarly, Riaz et al. (2022) suggested that SiNPs can relieve Cu^2+^ toxicity in wheat seedlings. SiNPs treated plants showed increased root length and plant height and enhanced antioxidant defence system. It was manifested by decreased malondialdehyde (MDA) and H_2_O_2_ contents and Cu^2+^ concentrations in shoots.

### 5.7 Manganese

Si-induced alleviation of Mn toxicity has been reported in several studies, suggesting Si could contribute to the depression of Mn uptake and transport (Li et al., 2015). It could restrict lipid peroxidation by upregulating non-enzymatic and enzymatic antioxidants (Shi et al., 2005; Li et al., 2015). The reduced ·OH accumulation was also detected in the leaf apoplast (Dragišić Maksimović et al., 2007, Dragišić Maksimović et al., 2012). Moreover, Doncheva et al. (2009) observed a substantial thickening of epidermal layers after the Si treatment in the Mn-sensitive maize variety over a tolerant one. It suggests that Si could induce Mn storage in non-photosynthetic tissue to prevent Mn-toxicity effects on chloroplast functions. Similarly, Iwasaki and Matsumura (1999) assume that Si could assist in displacing and storing Mn in a metabolically inactive form around the base of the trichomes on the leaf surface. The mitigative effect of Si in the form of nanoparticles on Mn-toxicity was also described in the aforementioned study (Emamverdian et al., 2020; see Chapter 5.6).

### 5.8 Zinc

Generally, Zn is a vital element for plant growth, as it is imperative in numerous metabolic pathways. Its deficiency is one of the plant’s most widespread micronutrient deficiencies (Anwaar et al., 2015; Kaur and Garg, 2021). However, as was reported by Long et al. (2003), a concentration above 3000 mg kg^−1^ Zn in dry soil can have a noxious effect on plant yield and growth as it alters the ionome of plants through the inhibition of nutrients’ uptake and translocation (Bokor et al., 2015). The potential utilisation of Si in alleviating Zn toxicity has been studied, for example, in cotton or bamboo species (Anwaar et al., 2015; Emamverdian et al., 2018a). Both studies suggested that Si can limit Zn bioavailability and instigate the plant defence system by increasing antioxidant capacity and non-enzymatic activity, thus alleviating cellular oxidative damage. Similarly, Song et al. (2014) described that Si activated and regulated some photosynthesis-related genes in *Oryza sativa* with high-Zn exposure, improving photosynthesis over Zn-stressed plants that lacked Si treatments. These studies operated with bulk-Si, whereas using the SiNPs could potentially support its beneficial effects. However, in highly Zn-polluted soils, Zn can coexist with silica in the form of a Zn-silicate complex (Goswami et al., 2022), and Bokor et al. (2015) revealed such a complex can have a comparable negative effect on Lsi gene expression and mineral nutrition homeostasis as high concentration of Zn alone.

### 5.9 Antimony

Sb is a non-essential metalloid with noxious effects for plants. However, studies to manage Sb-toxicity with SiNPs are rare. Still, there are several reports of Si-induced mitigation of Sb-toxicity in higher plants that could point in the right direction. Vaculíková et al. (2014, 2016) described Si-induced alleviation of Sb-toxicity on root growth and architecture in maize seedlings. Si supported the antioxidant defence system and thus reduced oxidative stress symptoms, and although Si did not reduce Sb content in roots, it considerably restricted Sb translocation to shoot. Shetty et al. (2021) attributed the blocked Sb translocation to root lignification, which was observed to a greater extent in Si-treated plants of *Arundo donax*. Moreover, enhanced photosynthetic pigments and overall photosynthetic yield were described. In poplar callus exposed to Sb-stress, Si declined the content of Sb in the calli and supported overall callus growth and nutrient uptake as well as the content of photosynthetic pigments. The improved Sb tolerance was secured via the Si-induced modification of antioxidant enzyme activity (Labancová et al., 2023). These findings also correspond to results obtained from the wild-type and the low-silica rice mutant cultivated with 10 or 30 μmol L^−1^ Sb (Huang et al., 2012). Si treatment promoted growth and decreased Sb content in the shoots of both mutants by regulating the Sb distribution between the roots and shoots.

### 5.10 Nickel

Although Ni is an essential component of several metalloenzymes of plants, it could be very toxic at supraoptimal concentrations. Like Sb, Ni-toxicity management is something least discussed in the literature with SiNPs. Exogenous Si was investigated in rice as a possible mitigative driver for Ni stress. Si protected the seedlings by upregulating the antioxidant defence components and glyoxalase systems, helping the ROS scavenge and detoxify cytotoxic methylglyoxal (Hasanuzzaman et al., 2013). The Si-induced recovery of growth, gas exchange, and pigment contents in cotton seedlings under the Ni stress was observed. It was secured by decreasing the Ni uptake and accumulation in the leaf, stem, and roots. It also increased antioxidant enzyme activities to restrict MDA, H_2_O_2_, and electrolyte leakage in leaves and roots (Khaliq et al., 2016). The enhanced antioxidant system, improved integrity of cell membranes and averted Ni-induced root anatomy alteration were also denoted as the mechanisms of Si-induced mitigation of Ni toxicity in maize plants (Vaculík et al., 2021). Improved leaf water status, enzymatic and non-enzymatic defence systems, and increased content of assimilatory pigments and leaf area were also described by Fiala et al. (2021).

## 6 Mechanisms underlying SiNPs-mediated defence responses

**Figure 3:**
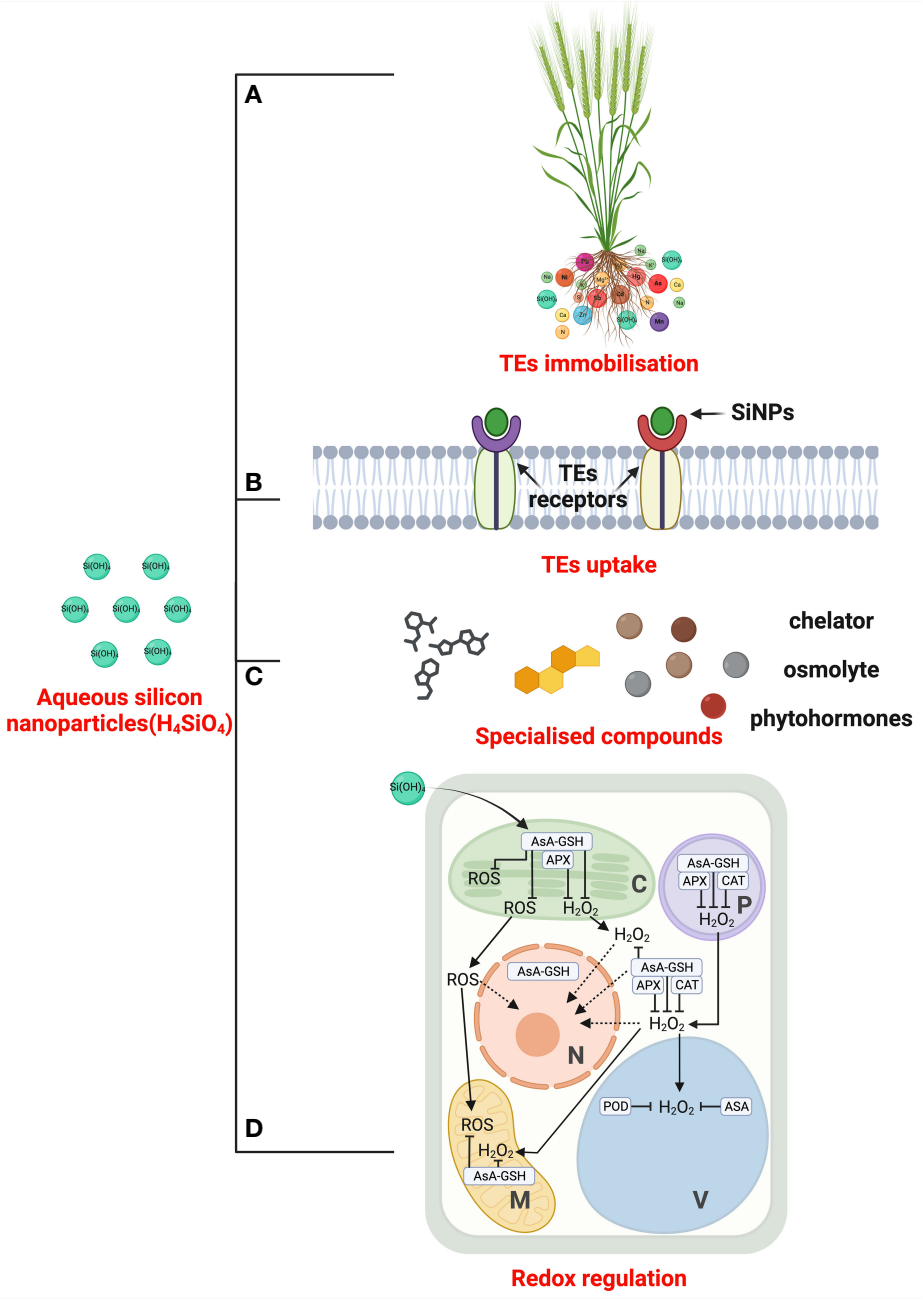
The mechanistic overview of silicon nanoparticles (SiNPs)-mediated TEs tolerance. SiNPs can increase the pH of the growing medium (soil and hydroponics) or alter elemental speciation by forming silicate complexes. It leads to TEs adsorption and immobilisation **(A)**. Further, the remaining TEs translocation is discouraged by blocking TEs receptors **(B)**. At inter- and intracellular spaces, SiNPs boost the biosynthesis of chelators compound, organic acid, and phenolic root exudation to minimise TEs toxicity **(C)**. SiNPs optimise redox status under TEs excess through supporting enzymatic and non-enzymatic antioxidant defence systems **(D)** and contribute to overall TEs tolerance in higher plants. TEs: trace elements; SiNPs: silicon nanoparticles; AsA: Ascorbic acid; GSH: glutathione; APX: ascorbate peroxidase; ROS: reactive oxygen species; H_2_O_2_: hydrogen peroxide; CAT: catalase; POD: peroxidase; alphabetic abbreviations inside panel **(D)** – C: chloroplast; P: Peroxisomes; N: Nucleus; V: Vacuole; M: mitochondria.

There are several mechanisms and pathways that SiNPs can adopt to mitigate TEs phytotoxicity. For instance, silicon can limit TEs uptake and translocation by lowering the ion activities in the medium. At a cellular level, it can regulate the co-precipitation of elements, antioxidant machinery, gene expression concerning TE transport and chelation, and morphological adjustments (Adrees et al., 2015; Emamverdian et al., 2018b). Here, we discussed the active processes of SiNPs against TEs toxicity (**Figure 3**).

### 6.1 TEs immobilisation in soil

Exogenous SiNPs ameliorate toxic TEs effects and improve overall plant growth (Tripathi et al., 2016; de Sousa et al., 2019; El-Saadony et al., 2021). These overcome responses have been correlated with soil physical properties, including TEs speciation, changes in soil pH, or deprivation of TEs due to co-precipitation with silicon (Bhat et al., 2019). Pretreatment of bulk-Si enhances phenolic root exudation (Chen et al., 2016) and organic acid exudation (Fan et al., 2016), which might be critical in TEs mitigation. Silicon immobilises TEs on the root’s outer surface by increasing the soil’s pH or altering TEs speciation by forming silicate complexes in soil solution. He et al. (2013) observed the complexification of most Cd with wall-bound silicon in rice root cells, leading to reduced Cd uptake and translocation. Kopittke et al. (2017) performed synchrotron studies and confined most of the Al complexed with Si in the *Sorghum bicolor* root cap. They evinced that this Si-Al complex formation in the root periphery could minimise the metal accumulation in the cell wall. Thus, the Si-mediated extrinsic defence mechanism through organic acid or phenolic exudation could be crucial in TEs toxicity mitigation.

### 6.2 Barrier to uptake and transport

Non-controlled silica deposition can harm a plant; hence, plants evolved effective mechanisms of cell walls’ silicification (Kumar et al., 2017). Silicon binds to lignin in the cell wall to form a Si-TE ion complex that subsequently arrests ion translocation from the root to the other plant organs (He et al., 2013; Sheng and Chen, 2020; Soukup et al., 2020). Most Si-mediated fruitful effects are reported to be linked with the accumulation of Si in roots, stems, leaves, and hulls, which acts as a physical barrier by enhancing the mechanical strength of plant tissues (Ma et al., 2006; Emamverdian et al., 2018b). Si, along with lignin, can deposit in dermal regions of the cell walls, thickening the Casparian strips and blocking the TE transport in plants (Kim et al., 2014). Si-induced changes in the cell wall binding properties might be essential in mitigating TEs toxicity. Si reduced Cd accumulation in roots and grains of rice (Chen et al., 2019) and maize (Liu et al., 2020b) which could result from Si deposition in root cells apoplast hindering Cd uptake (Song et al., 2009; Wang et al., 2015). It is stipulated that Si enhances Lsi expression while suppressing Nramp 5 (Cd transporter gene) in rice roots, suggesting that upregulated Si transporters resist Cd toxicity under ample silicon supply (da Cunha and do Nascimento, 2009; Ma et al., 2015). Considerable amounts of covalent-bound Si are also complexed with hydroxyl groups of pectin contained in the cell wall (Schwarz, 1973; Sheng and Chen, 2020). He et al. (2015) suggests that hemicellulose, rather than pectin and cellulose, is the primary ligand bound to Si complexes in rice. Si accumulated in the cell walls in hemicellulose-bound organosilicon compounds can improve cell wall mechanical properties and regeneration and inhibit the Cd uptake by a mechanism of Cd complexation and subsequent co-deposition (He et al., 2015; Ma et al., 2015).

### 6.3 Active participation in the antioxidant defence system

TEs overaccumulation stages the ROS-induced oxidative emergency, threatening many vital processes. Thus, the plant’s top priority for survival under such scenarios is scavenging ROS. This goal is facilitated by an antioxidant system comprising several enzymatic (SOD, CAT, APX, POD, and GR) and non-enzymatic (ascorbic acid, α-tocopherol, proline, carotenoids, flavonoids, and reduced glutathione) antioxidants. In this context, pretreatment of SiNPs was reported to stimulate the enzymatic antioxidants against TEs toxicity in *Solanum lycopersicum* under As stress (González-Moscoso et al., 2019, González-Moscoso et al., 2022), *Glycine max* under Hg stress (Li et al., 2020), and *Satureja hortensis* (Memari-Tabrizi et al., 2021) and *Triticum aestivum* under Cd toxicity (Ali et al., 2019). The formation of free radicals under TEs toxicity directly damages the cell membrane permeability and stability that, in time, would cause the homeostasis collapse of cells and tissues. However, silicon counteracts it by enhancing the stability of the plasma membrane under TEs stress (Vaculík et al., 2020). Another TEs detoxifying mechanism is the synthesis of various chelating agents, i.e., flavonoids, phenolics, and organic acids (Bhat et al., 2019). Silicon reportedly influences the synthesis of several chelating compounds, including cysteine, glutathione, and phytochelatins, under TEs toxicity (Rahman et al., 2017).

## 7 ‘Omics’ bases of SiNPs-induced TEs sequestration

### 7.1 Metabolomics

Plants evolved several responsive manoeuvers against TEs toxicity, such as minimising TEs bioavailability and uptake, enriching plants with nutrients, and stimulating the antioxidant system and the biosynthesis of protective agents (osmolytes, organic acids, metallothioneins, and phytochelatins) (Tripathi et al., 2015; Moharem et al., 2019; Cao et al., 2020; Lian et al., 2020; Wang et al., 2020a, Wang et al., 2020b). Among the different NPs applied, SiNPs have proven to be quite promising (Tripathi et al., 2015; Wang et al., 2015; Tripathi et al., 2017; Khan et al., 2020a). Though not an exclusively effective metal/metalloid barricade, the apoplasmic barrier also fulfils essential defensive functions in plant roots by regulating the flow of ions, oxygen and water (Lux et al., 2004; Chao et al., 2013). The efficiency of apoplasmic barriers as contaminant barricades can be enhanced by NPs application (Rossi et al., 2017). NPs attach to the TEs in the root cell walls, making stable complexes and rendering them unavailable. NPs-TEs complexes, once adsorbed, become immobile, obstructing the mobility of the TEs inside the plants and reducing their biological activity (Cui et al., 2017; Wang et al., 2021; Zhou et al., 2021). Accumulating organic acids (behaving as metal chelators) and chelating TE contaminants are necessary adaptations to TEs tolerance. The biosynthesis of such protective organic acids is improved by SiNPs, reducing the damage caused by TEs like Cd and As (Cui et al., 2017; Tripathi et al., 2017; Zhou et al., 2021). The interaction of SiNPs with the TEs is crucial while studying the different characteristics of TEs stress alleviation. SiNPs can also reduce the mobility and bioavailability of TE contaminants in the soil (Tripathi et al., 2015; Wang et al., 2015; Tripathi et al., 2017; Khan et al., 2020a). The application of mercapto SiNPs increased the stability of Cd and, thus, decreased its mobility (Wang et al., 2020b). Alternatively, Si may co-precipitate with metals/metalloids as silicates in the roots and leaves of different plants. Co-precipitation of Zn as Zn silicates or Si–Zn complexes in the cell walls of leaf epidermal cells was seen in *Minuartia verna* and *Cardaminopsis halleri* (Neumann et al., 1997; Neumann and Zur Nieden, 2001). Gu et al. (2011, 2012) suggested the sequestration of Zn–Si and Zn-Cd precipitates in rice to the cell walls of less bioactive tissues. Similarly, Mn, Cu, and Cd might co-precipitate with Si to restrict their accumulation in shoot phytoliths, but it is not fully confirmed yet (Iwasaki et al., 2002; Zhang et al., 2008; Oliva et al., 2011; Emamverdian et al., 2020).

SiNPs regulate a variety of physiological phenomena in plants, notably nutrient assimilation, CO_2_ fixation, accretion of secondary metabolism products and activities of different enzymes under normal as well as perturbed environmental conditions (Tripathi et al., 2015, Tripathi et al., 2017; Ahmad et al., 2019; Khan et al., 2020a; Mukarram et al., 2021). Al-toxicity alleviation in barley and maize after Si supplementation has been endorsed to accumulate phenolic compounds (Adrees et al., 2015; Vega-Mas et al., 2019). SiNPs have also been observed to modulate the regulatory enzymes of the shikimic acid pathway, leading to increased accretion of phenols in the leaves, as has been reported in *Mentha piperita* (Ahmad et al., 2019). Because of their metal-chelation capacity with flavonoid-phenolics, phenols play a critical role in TE toxicity mitigation by reducing the uptake and translocation of toxic TEs, as has been reported for Al and Mn (Kidd et al., 2001; Dragišić Maksimović et al., 2007; Shahnaz et al., 2011). Bulk-Si facilitates phenol overaccumulation by triggering phenylalanine ammonia-lyase (PAL), a critical regulatory enzyme of the phenylpropanoid pathway (Rahman et al., 2015; Ahanger et al., 2020). It also upregulated PAL, cinnamyl alcohol dehydrogenase, and chalcone synthase in *Rosa hybrida* (Shetty et al., 2011). Likewise, Si-induced PAL activity also assisted in managing Cu stress in *Arabidopsis thaliana* roots (Li et al., 2008).

Bulk-Si can influence the biosynthesis of H_2_O_2_, nitric oxide (NO), and hydrogen sulphide (H_2_S) to govern Ag and Cd toxicity in mustard and pepper (Soundararajan et al., 2018; Vishwakarma et al., 2020; Kaya et al., 2020a). Si decreases electrolyte leakage and H_2_O_2_ and MDA content by boosting the antioxidant system, possibly with NO involvement, and regulates plant growth and development under TE toxicity (Tripathi et al., 2021). SiNPs-induced NR biosynthesis in *Mentha piperita* and restricted H_2_O_2_ production in *Cymbopogon flexuosus* suggests SiNPs crosstalk with H_2_O_2_ and NO (Ahmad et al., 2019; Mukarram et al., 2023). Thus, it does not seem hasty speculation that SiNPs could interact with gaseous signalling molecules in a similar fashion to bulk-Si. It will hold relevance during TEs stress alleviation as well. The nitrate reductase (NR) pathway is the best-characterised NO biosynthetic pathway (Planchet and Kaiser, 2006). In addition to the general function of nitrate-to-nitrite reduction, NR also performs a crucial part in plants by transferring an electron to nitrite using NAD(P)H as a source of electrons, ultimately resulting in NO biosynthesis (Planchet and Kaiser, 2006). The synergistic interaction of NO and Si can discourage As uptake and increase phytochelatin biosynthesis, reducing As translocation in mustard (Ahmad et al., 2021). In a similar study, Liu et al. (2020b) established the collegial effect of Si and NO in mitigating Cd toxicity in *Triticum aestivum* seedlings. Si stimulates endogenous H_2_S accretion that upregulates antioxidants’ activity in *Capsicum annuum* raised on Cd- and B-spiked soils (Kaya et al., 2020a, 2020b).

### 7.2 Proteomics

Given the scanty literature concerning the SiNPs-mediated TEs tolerance in plants, the proteomic approach is comparatively novel to gaining insights into the expression of various stress-related enzymes and proteins. TEs-stimulated oxidative stress leads to altered protein expression and structure, leading to loss of protein activity or its content. Nevertheless, silicon and SiNPs supplementation regulates the expression of several proteins and enzymes of signal transduction cascades of the antioxidant defence system (Tripathi et al., 2015; Muneer and Jeong, 2015a; Mukarram et al., 2022). Once inside the cells, silicon plays an imperative role in stress alleviation by maintaining ion homeostasis and structural rigidity, upregulating antioxidant metabolism, and increasing the expression of genes and proteins involved in stress alleviation (Ma, 2004). SiNPs-induced upregulation of the different primary and secondary metabolic enzymes is well-reported. Mukarram et al. (2021) reported that the expression of terpene (neral, geranial) and NR enzyme activity was upregulated in SiNPs-treated *Cymbopogon flexuosus*. Si application improves PSII polyprotein expression under Zn toxicity (Song et al., 2014). Further, the bulk-silicon enhanced the protein content related to stress (17%), hormones (11%), and other cellular biosynthesis (11%), and many others associated with gene expression and secondary metabolism in 25 mM salt-stressed *Lycopersicon esculentum* plants (Muneer and Jeong, 2015b). The stress-related proteins included zinc finger A20, COPINE 1 family protein, caffeoyl-CoA O-methyltransferase, and others. Down-regulation of Zn transporter (*OsZIP1*) protein after Si supplementation decreases Zn uptake in *Oryza sativa* (Huang and Ma, 2020). Si accumulation involves both influx and efflux transporters. The SiNPs application has been endorsed for upregulating the menthol-reductase enzyme to proliferate menthol in mint oil (Ahmad et al., 2019). Similarly, geraniol dehydrogenase enzyme activity was positively influenced by SiNPs foliar application in *Cymbopogon flexuosus* under 160 mM and 240 mM salt stress (Mukarram et al., 2023). SiNPs-induced antioxidants can improve isoenzyme patterns and genomic alterations to restrict TE toxicity in *Pisum sativum* and UV-B stress in *Triticum aestivum* (Tripathi et al., 2015, Tripathi et al., 2017). Further, Si can delay chlorophyll-protein complex degradation such as supercomplexes, PSI core binding LHCI, PSI core, F1-ATPase binding Cytb6/f complex, PSII core, and trimeric and monomeric LHCII (Wang et al., 2019). Si can also improve photosynthetic performance, given its observed benefits on absorption, transformation, and transfer of light energy through optimising thylakoid membrane proteins in water-deprived *Oryza sativa* seedlings (Wang et al., 2019).

### 7.3 Genomics

Studies on TEs-stressed plants have indicated that ROS-induced DNA damage was more pronounced in the Cd and Pb-exposed plants, as indicated by the disappearance of several normal bands in the RAPD pattern of the DNA. In contrast, new DNA amplicons could be located in TE-exposed plants treated with different NPs. Moreover, oxidation of proteins is a common TEs toxicity symptom as TEs ions directly interact with protein molecules due to their strong affinity with carboxyl- thionyl- and histidyl groups (Hossain et al., 2015). Studies have revealed that the NPs within the plant cell systems may interact with these sulfhydryl and carboxyl groups, eventually altering the protein activity by acting and reacting similarly to the metal ions (Hossain et al., 2015). As discussed, different NPs upregulate the expression of various genes in plants, speeding up the biosynthesis of several primary and secondary metabolism products (Večeřová et al., 2016; Marslin et al., 2017).

The role of SiNPs exemplifies regulating an array of transcription factors (TFs) implicated in abiotic mitigation, notably DREB2, NAC, NAM, and CUC. These TFs overexpress genes associated with scavenging free radicals and maintaining osmotic potential and ionic homeostasis (Manivannan and Ahn, 2017). Moreover, silicon can induce regulatory proteins coupled to gene expression under stress, particularly TFs for transcription elongation (SPT4), ribosomal protein L16, RNA polymerase mediator, tRNA-lysidine synthase, MADS-box, ribosome-recycling factor and reverse transcriptase (Muneer and Jeong, 2015b; Al Murad et al., 2020).

Exogenous application of SiNPs modifies plant’s nutrient status, facilitating N, Fe, Mg, Zn, and Si absorption (Wang et al., 2015; Ahmad et al., 2019; Mukarram et al., 2021). SiNPs-mediated increase in Si uptake leads to a decrease in the Cd uptake, facilitating the growth of *Oryza sativa* seedlings raised on Cd-rich soils. Cui et al. (2017), in their study on rice, observed that SiNPs upregulate the expression of Si transporter (*OsLsi1*) while the expression of Cd-transporters (*OsLCT1, OsNramp5*) is down-regulated. Down-regulation of essential abiotic stress tolerance genes, notably *ERF5* (ethylene response factor 5, *RBOH1* (respiratory burst oxidase), *MAPK2, and MAPK3* (mitogen-activated protein kinases), by the application of SiNPs, is well-reported (Almutairi, 2016). SiNPs-mediated regulation of primary metabolism, biosynthesis and modifications of secondary metabolism products, particularly phenols, possibly enhances the tolerance against stress (Rahman et al., 2015; Tripathi et al., 2015, Tripathi et al., 2017; Ahmad et al., 2019; Ahanger et al., 2020). Furthermore, SiNPs were suggested to induce different transcripts (*CfADH2a*-b, *CfADH1, CfAKR2b, CfAAT3*, and *CfALDH*) to regulate the constitutional makeup of plant essential oil (Mukarram et al., 2021).

Despite growing studies on SiNPs, most publications need more genomic insights. Here we introduce significant recent findings dealing with the influence of Si on gene activity, generally to indicate a possible role with SiNPs. Different studies have suggested the positive regulation of gene transcripts of various metabolic processes by Si application (Brunings et al., 2009; Chain et al., 2009; Debona et al., 2017). The role of Si in upregulating photosynthetic genes has been studied in detail. For example, Zn-induced damage in *PsbY* expression was overcome by Si supplementation (Song et al., 2014). Moreover, increased PSII activity and electron transfer rate by upregulating *PsbY* mRNA transcripts are endorsed for Si supplementation in rice under Zn stress. The other Si-upregulated genes under Zn-toxicity include *PetC, PsaH, PetH* encoding chloroplast, cytochrome proteins, and ferredoxin NADP^+^ reductases, respectively (Song et al., 2014). Moreover, Si upregulates several genes encoding for electron transport chain proteins and light-harvesting complex viz., *PetE*, *PetF*, *PsbQ, PsbP*, *PsbW, and Psb28*. Furthermore, increased expression of gene transcripts (*PsbW, Psb28, PsbQ, and PsbP*) involved in the photolysis of water has been attributed to Si application (Zhang et al., 2018). *PetH*, *Os03g57120* and *Os09g26810* genes involved in stress mitigation, NAD(P)H and glutathione biosynthesis are also upregulated by Si (Manivannan and Ahn, 2017). Kaushik and Saini (2019) have reported the upregulation of *LeGR* (glutathione reductase gene) in *Solanum lycopersicum* after Si supplementation. In *Triticum aestivum*, Si attenuates TEs toxicity by upregulating metallothionein and phytochelatin synthase gene expression (*TaMT1*, *TaPCS1*) (Hossain et al., 2018). Genes coding for enzymatic oxidants (*SlCAT*, *SlGR*, *SlGST*, *SlSOD*, *SlPOD*, *SlGPX*) have been observed to be upregulated by exogenous sourcing of Si attenuating stress response in *Solanum lycopersicum* (Khan et al., 2020b).

## 8 Conclusion and future trends

Only a few published articles have focused on the interaction between SiNPs and TEs toxicity, with existing reviews mainly discussing heavy metals and neglecting other toxic elements. These reviews also fail to emphasise SiNPs over bulk silicon or adequately address the omics aspect. In our previous review (Mukarram et al., 2022), we explored SiNPs potential in alleviating abiotic stress, including metals stress, but needed TEs and a detailed *modus operandi*. To address these concerns, our present review includes a wide range of toxic elements studied with SiNPs, regardless of the ‘heavy metals’ label, and explores how SiNPs interact with plant metabolomics, proteomics, and genomics during TEs toxicity. The novelty of our review lies in its understanding of the SiNPs-TEs interaction and its omics perspective, aiming to stimulate a discussion within the silicon community about its active involvement (if any) in plant physiology, particularly given the existing uncertainties in this field.

In the present review, we focused mainly on the action of SiNPs during TEs presence. It could be understood from several studies that SiNPs have superior benefits against TEs excess over bulk-Si. The *modus operandi* relies upon SiNPs-induced chelation and immobilisation of TEs at the first contact site, i.e., soil. Once toxic elements are inside the plant, SiNPs might compartmentalise TEs or restrict them in vacuoles and cell walls. SiNPs further attenuate TEs stress by inducing biochemical defence such as antioxidants, osmolytes, and other specialised compounds. Several proteins and genes have been identified to support SiNPs action in TEs-stressed plants. Nonetheless, future studies could address the following concerns:

1. Much prospective research is encouraged on SiNPs interaction with TEs toxicity.
2. It is high time to produce empirical proof of whether SiNPs provide more anatomical and structural support or physiological participation during TEs toxicity.
3. More research is required for SiNPs action on certain (less-discussed) trace elements such as Mo and Se.
4. A lack of omics approaches in contemporary studies is still prevalent.
5. The aquaporins for TEs uptake and distribution need to be identified and sequenced.
6. Silicon channels are needed for other model plant species, especially C4 plants, such as sorghum and sugarcane where silica can be stored at higher concentrations.

## Author contributions

MM: Conceptualization, Project administration, Resources, Supervision, Visualization, Writing – original draft. BA: Writing – original draft. SC: Writing – original draft. AK: Writing – original draft. DK: Funding acquisition, Project administration, Writing – original draft, Writing – review & editing. MK: Project administration, Supervision, Validation, Visualization, Writing – review & editing. AL: Project administration, Supervision, Validation, Visualization, Writing – review & editing.
